# A Thickening Agent Using *Dioscorea japonica* Powder Exhibits Suitable Properties for People with Dysphagia

**DOI:** 10.3390/foods12213943

**Published:** 2023-10-28

**Authors:** Yuka Konoike, Izumi Tsukayama, Mei Oji, Takayo Kawakami, Kayoko Ishii, Toshiko Suzuki-Yamamoto

**Affiliations:** 1 Department of Nutritional Science, Okayama Prefectural University, 111 Kuboki, Soja, Okayama 719-1197, Japan; konoike@fukuyama-u.ac.jp (Y.K.); tsukayama@fhw.oka-pu.ac.jp (I.T.); mei.ouji@ksu.ac.jp (M.O.); kawakami@fhw.oka-pu.ac.jp (T.K.); 2 Department of Nutrition and Life Science, Fukuyama University, 985-1 Sanzo, Higashimura-cho, Fukuyama 729-0292, Japan; ishii@fukuyama-u.ac.jp; 3 The Faculty of Food Culture, Kurashiki Sakuyo University, 3515 Tamashima Nagao, Kurashiki, Okayama 710-0292, Japan

**Keywords:** dysphagia diet, *Dioscorea japonica*, thickened liquid, viscosity, functional food

## Abstract

In an aging society, the novel concept of added food functionality in a dysphagia diet is necessary for preventing diseases and maintain nutrition intake. The present study evaluated the utilization of *Dioscorea japonica* as a thickened liquid for people with dysphagia due to its unique physical properties and beneficial effects on chronic inflammation. The viscosity of the prepared thickened liquid using freeze-dried *Dioscorea japonica* powder was compared with those of xanthan gum and commercially available thickened liquids in selected conditions resembling to cooking. *Dioscorea japonica* powder showed high versatility, because the viscosity of its thickened liquid could be easily adjusted by modifying its blending amount and temperature. The thickened liquid of *Dioscorea japonica* had the most stable viscosity among the thickened liquids when NaCl was added and exhibited excellent resistance to α-amylase, similar to that of the other thickened liquids. The viscosity of the thickened liquid of *Dioscorea japonica* was relatively stable on changing the pH, but it was slightly unstable when the temperature changed. Overall, the thickened liquid of *Dioscorea japonica* powder has excellent viscosity stability, comparable to or better than commercially available thickened liquids, and is expected to be used as a new thickened liquid with added food functionality.

## 1. Introduction

Chronic diseases including malignant neoplasms, heart diseases, and cerebrovascular diseases, which are the main causes of death in Japan, are in many cases associated with chronic inflammation. Thus, the prevention of chronic inflammation is the most important factor in maintaining a healthy and long-living society. For the prevention of chronic inflammation, we have explored food functionality targeting the synthetic pathway of ω-6 arachidonic acid-derived prostaglandin (PG) E_2_, which induces various inflammatory diseases, that is, it is called a proinflammatory lipid mediator. Previously, we demonstrated that a wild yam *Dioscorea japonica* belonging to the *Dioscoreaceae* family suppressed cyclooxygenase-2 (COX-2) and microsomal prostaglandin E (PGE) synthase-1, which are key enzymes in the production of proinflammatory PGE_2_, and *Dioscorea japonica* exhibited anti-inflammation and anti-carcinogenesis [1,2]. Diosgenin, a critical functional ingredient of *Dioscorea japonica*, downregulated COX-2 expression selectively in macrophages via the glucocorticoid receptor and improved LPS-induced mouse liver inflammation [3]. Thus, we have confirmed that *Dioscorea japonica* and the functional ingredient diosgenin led to a decrease in PGE_2_ production and improvement in chronic inflammatory diseases in vitro and in vivo.

*Dioscorea japonica* is a relative of the *Dioscoreaceae* family, which is native to Japan. The yam tubers of this species are usually considered edible and are beneficial for nutritional fortification [4,5]. Moreover, they have been shown to have other beneficial effects such as gastric mucosal protection and digestive enhancement. In some countries, including Japan, the wild yam has been used as folk medicine against asthma, rheumatoid arthritis, bronchitis, and other diseases [6,7,8]. *Dioscoreaceae* yam tubers have distinguishing physical properties, and among them, *Dioscorea japonica* has the highest viscosity. Previously, we elucidated the rheological properties of *Dioscorea japonica* paste and its potential application in dysphagia diets [9]. It was demonstrated that the paste prepared from the freeze-dried *Dioscorea japonica* powder (30% (*W*/*V*)) had a suitable texture profile of hardness, cohesiveness, and adhesiveness, and complied with a level II dysphagia diet as required by the Japanese Ministry of Health, Labour and Welfare. In addition, our rheological analysis [9] indicated that the rheological profile of *Dioscorea japonica* paste (20–30% (*W*/*V*)) was more suitable as a dysphagia thickened liquid than grated raw *Dioscorea japonica* and was similar to that of a true polymer solution, xanthan gum 0.2% (*W*/*V*) [10]. These results suggested that the *Dioscorea japonica* powder has a potential application in dysphagia diets. Furthermore, texture profile analysis indicated that *Dioscorea japonica* paste showed a similar physical property and swallowing compatibility to yogurt, which is often used for swallowing training for patients with dysphagia [11].

In Japan, a super-aged society, patients with dysphagia are increasing [12,13,14]. Dysphagia leads not only to an impaired quality of life and malnutrition but also severe diseases such as aspiration pneumonia [15,16]. To maintain a healthy body and activities of daily living in the elderly, it is essential to take high-quality nutrition orally. In patients with impaired swallowing function, consumption of texture-modified food and thickened liquids is preferable, and therefore, commercial thickening and gelling agents are commonly used for them. Several studies have shown that using thickening agents has a positive effect on swallowing [17,18]. In addition, around the world, standards have been established to ensure the appropriate use of texture-modified foods and liquids for people with dysphagia [19,20,21]. Today, xanthan-gum-based thickened liquids are mainly used to prepare foods that vary in texture and for patients with dysphagia for ease of swallowing [22,23]. It is important to select an appropriate thickened liquid with rheological properties based on the individual swallowing function to avoid the risk of aspiration. The characteristics of *Dioscorea japonica* regarding food functionality and physical properties could be beneficially applied in a novel dysphagia diet [1,2,9]. There have been only a few studies on the application of thickening agents with added food functionality [24,25], and there is no study comparing these with commercially available thickening agents except the present study. Then, the stability of the physical properties during cooking and food processing will need to be confirmed. In the present study, we compared the viscosity of the prepared thickened liquid of *Dioscorea japonica* (*Dioscorea japonica* liquid) with that of xanthan gum and commercially available thickened liquids under several conditions such as temperature, pH, and the addition of NaCl or α-amylase.

## 2. Materials and Methods

### 2.1. Sample Preparation

*Dioscorea japonica* powder, which is a commercially available freeze-dried fine powder that can be used for cooking, was purchased from Naturalskyway Co. (Tokyo, Japan). It was mixed with distilled water or 20 mM sodium phosphate buffer (pH 7.0) at 20 °C ± 2 °C using an electric mixer (bamixM250, ESGE Ltd., Mettlen, Switzerland) at room temperature for 5 min. The uniformly mixed *Dioscorea japonica* powder was kept as is for 20 min to remove any bubbles that were formed. Commercially available thickening agent A (TROMELIN V) containing dextrin, thickening polysaccharide, and potassium chloride was purchased from NUTRI Co., Ltd. (Yokkaichi, Japan); commercially available thickening agent B (TSURURINKO Quickly) containing dextrin, xanthan gum, calcium lactate, and trisodium citrate was purchased from CLINICO Co., Ltd. (Tokyo, Japan); and xanthan gum was purchased from Taiyo Kagaku Co., Ltd. (Yokkaichi, Japan). They were added to distilled water or 20 mM sodium phosphate buffer (pH 7.0) at 20 °C ± 2 °C and stirred manually with a spatula approximately 60 times/min for 1 min. The thickened liquids were stirred manually 10 times before the viscosity analysis. As the solvent, sodium phosphate solution was used for the effect of NaCl addition, and water was used for others.

### 2.2. Viscosity Analysis

Viscosity of the thickened liquids was analyzed according to the method established by JDD2013 (the Japanese Society of Dysphagia Rehabilitation (JSDR) dysphagia diet committee) [21], using a cone and plate viscometer (VISCOMETER TV-25; Toki Sangyo, Co., Ltd., Tokyo, Japan) with a cone diameter of 28 mm and angle of 3°. Viscosity of all thickened liquids was measured after 1 min under the following setting: temperature of 20 °C and shear rate of 50 s^−1^.

### 2.3. Line Spread Test

The line spread test (LST), another simple viscosity test established by JDD2013 [21], was performed using a flat plate with concentric circles (Saraya Co., Ltd., Osaka, Japan). A hollow ring with an internal diameter of 30 mm and height of 28 mm was filled with 20 mL of the thickened liquid and was left to stand for 30 s. The hollow ring was then lifted, and the sample was allowed to spread for 30 s. The distances covered by the liquid along the six axes were read and averaged [26]. This test was performed three times each, and each value was averaged and evaluated.

### 2.4. Effect of Temperature on the Viscosity of the Thickened Liquids

The prepared samples were kept for 20 min at 10, 20, 40, or 60 °C by water bath before conducting the viscosity analysis. All samples were adjusted to 150–300 mPa∙s as moderately thick according to JDD2013 by the JSDR dysphagia diet committee [21].

### 2.5. Effect of pH on the Viscosity of the Thickened Liquid

After preparation of the thickened liquids, pH of all samples was changed by addition of the following buffers (final concentration of 20 mM): sodium acetate buffer (pH 4.0), sodium phosphate buffer (pH 7.0), and glycine buffer (pH 9.0).

### 2.6. Assessment of α-Amylase

After preparation of the thickened liquids, they were incubated with α-amylase (1–1000 μg /mL, Wako Pure Chemical Industries, Ltd., Osaka, Japan) in 20 mM sodium phosphate buffer (pH 7.0) at 37 °C for 10 min. The thickened liquid prepared using potato starch (5.3%) was used for comparison. Their viscosities were then measured at 20 °C for 1 min, as previously described.

### 2.7. Statistics

Data were statistically evaluated through ANOVA, using Bonferroni test or Dunnett’s test and a significance level of *p* < 0.01. All statistical analyses were performed using the KaleidaGraph Win 4.5 (Synergy Software, Reading, PA, USA).

## 3. Results

### 3.1. The Correlation between the Concentration and Viscosity of Dioscorea japonica Liquid and Thickened Liquids

**Figure 1:**
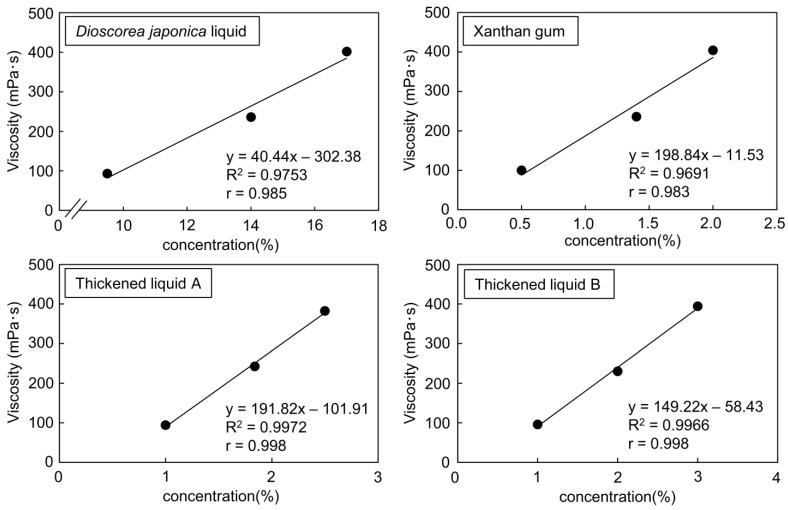
Relationship between concentration and viscosity in each sample. *Dioscorea japonica* liquid was evaluated at concentrations of 9.5%, 14%, and 17%. Xanthan gum was evaluated at concentrations of 0.5%, 1.4%, and 2.0%. Thickened liquid A was evaluated at concentrations of 1.0%, 1.8%, and 2.5%. Thickened liquid B was evaluated at concentrations of 1.0%, 2.0%, and 3.0%. The solutes were dissolved in distilled water. The viscosity of all samples was assessed using a viscometer at 20 °C after five independent measurements.

Several commercially available thickened liquids have individual physical properties, with different viscosities and textures even at the same concentration [27,28]. The correlation between concentration and viscosity of the *Dioscorea japonica* liquid, liquid xanthan gum, and commercially available thickened liquids A and B was confirmed (Figure 1). Table 1 shows the comparative concentration of each thickener based on the three-stage classification by the established standard viscosity of JDD2013, “mildly thick”, “moderately thick”, and “extremely thick” [29]. The *Dioscorea japonica* liquid (9.5%, 14%, and 17%), liquid xanthan gum (0.5%, 1.4%, and 2.0%), thickened liquid A (1.0%, 1.8%, and 2.5%), and thickened liquid B (1.0%, 2.0%, and 3.0%) were prepared to the median of each standard viscosity. Their viscosities and LST values were measured, and whether they matched with the criteria established by JDD2013 was evaluated (Table 2). All the samples were matched with the criteria of the viscosities and LST values established by JDD2013. Because there were some samples that showed a significant difference compared with *Dioscorea japonica* in the “mildly thick” category, the samples of thickened liquid adjusted to “moderately thick” were used in the subsequent experiments.

### 3.2. The Effect of Temperature Changes on the Viscosity of Thickened Liquids

**Figure 2:**
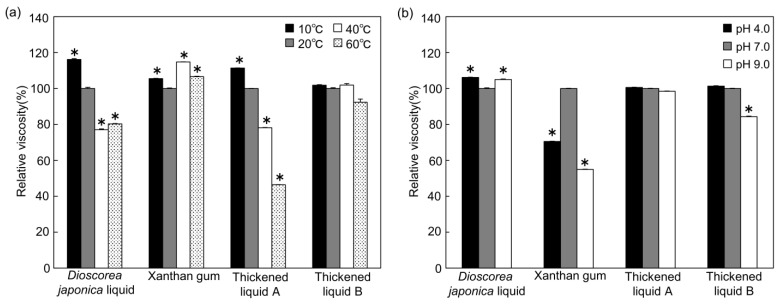
Effects on stability after temperature and pH changes to viscosity for each sample. (**a**) The viscosity of each sample was measured at 10, 20, 40, and 60 °C using a viscometer after five independent measurements. The solutes were dissolved in distilled water. (**b**) Each sample was prepared with sodium acetate buffer, sodium phosphate buffer, and glycine buffer at pH 4.0, 7.0, and 9.0, respectively. Sample concentration: *Dioscorea japonica* liquid 12% (**a**) and 13.5% (**b**), xanthan gum 0.7%, thickened liquid A 1.8%, thickened liquid B 2.1%. The values of relative viscosities are means ± SE represented as relative values with respect to the amount at 20 °C (**a**) or pH = 7.0 (**b**) as 100%. * *p* < 0.01 compared with 20 °C (**a**) or pH = 7.0 (**b**).

Changes in the viscosity of the thickened liquids, *Dioscorea japonica* liquid, liquid xanthan gum, thickened liquid A, and thickened liquid B (“moderately thick”, 229–242 mPa·s) were measured under various temperature conditions (10, 20, 40, and 60 °C) (Figure 2a). With respect to a temperature change, thickened liquid B was extremely stable without a significant difference, and the liquid xanthan gum was relatively stable with an increase of less than 14% at each temperature. In contrast, the viscosity of the *Dioscorea japonica* liquid increased by 16% at 10 °C and decreased by approximately 20% at 40 °C and 60 °C. Thickened liquid A was more likely to be affected by a change in temperature than the *Dioscorea japonica* liquid, and the viscosity of thickened liquid A increased by 11% at 10 °C and decreased by 22% and 54%, respectively, at 40 °C and 60 °C.

### 3.3. The Effect of pH Changes on the Viscosity of Thickened Liquids

The effect of a change in pH (pH 4.0, 7.0, and 9.0) on the viscosity of the thickened liquids was investigated (Figure 2b). Thickened liquid A was stable under the testing pH conditions. The viscosity of the *Dioscorea japonica* liquid marginally increased (within 7%) at pH 4.0 and 9.0 compared with pH 7.0. The viscosity of thickened liquid B showed a decrease of 16% at pH 9.0. Among the viscosities of all the thickened liquids, that of liquid xanthan gum was the most unstable in terms of a change in pH for pH 4.0 and 9.0, showing significant decreases of 30% at pH 4.0 and 45% at pH 9.0.

### 3.4. The Effect of NaCl Addition on the Viscosity of Thickened Liquids

**Figure 3:**
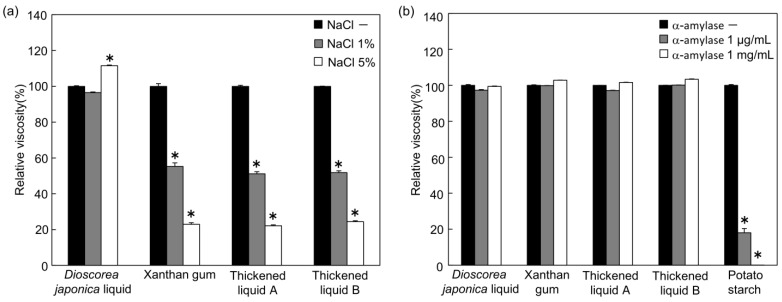
Effects on stability after NaCl addition and resistance on α-amylase activity to viscosity of each sample. (**a**) In each sample (pH = 7.0), 0%, 1%, or 5% NaCl in 20 mM sodium phosphate buffer was added. The viscosity of all samples was measured using a viscometer at 20 °C after five independent measurements. (**b**) Potato starch was used at a concentration of 5.3%. In each sample, or potato starch as a positive control, 0, 1, or 1000 μg/mL of α-amylase was added. The solutes were dissolved in distilled water. The viscosity of all samples was measured using a viscometer at 20 °C after five independent measurements. Sample concentration: *Dioscorea japonica* liquid 15% (**a**) and 12% (**b**), xanthan gum 0.7%, thickened liquid A 1.8%, thickened liquid B 2.1%. The values of relative viscosity are means ± SE represented as relative values with respect to the amount without NaCl (**a**) or α-amylase (**b**). * *p* < 0.01 compared with 0% NaCl (**a**) or 0 μg/mL α-amylase (**b**).

Because thickening agents are frequently added to foods that contain salt, the effect of NaCl addition (1% and 5%, *W*/*V*) on the viscosity of the thickened liquids was investigated (Figure 3a). A suitable NaCl concentration for soups or other relevant food items is approximately 0.9%, with 5% NaCl usually considered as being outside the normal accepted range. Among all the thickened liquids, the viscosity of the *Dioscorea japonica* liquid was the most stable following NaCl addition, although its viscosity slightly increased (12%) with the addition of 5% NaCl. In contrast, the viscosities of the liquid xanthan gum and thickened liquids A and B significantly decreased by approximately 50% and 80% with the addition of 1% and 5% NaCl, respectively.

### 3.5. Resistance of the Viscosity of Thickened Liquids to α-Amylase Addition

Starch-based thickeners are also commonly used in the management of dysphagia; however, the viscosity is unstable against α-amylase. Therefore, many commercially available thickening agents with resistance to salivary digestion are used. To examine the direct effect of α-amylase on the viscosity of thickened liquids, the resistance of the *Dioscorea japonica* liquid to α-amylase was compared with the resistance of other thickened liquids to α-amylase in the viscosity analysis (Figure 3b). The addition of α-amylase (1 μg/mL and 1 mg/mL) significantly decreased the viscosity of the potato starch liquid to 18% and 0.02%, respectively, after 10 min. In contrast, the viscosity of the *Dioscorea japonica* liquid was extremely resistant to α-amylase, which was similar to those observed for liquid xanthan gum and commercially available thickened liquids.

## 4. Discussion

In Japan, the most dominant standard for thickened liquids is formulated by JDD2013 and categorized on the basis of viscosity. In this study, we compared the usefulness of the thickened liquid of *Dioscorea japonica* with that of commercially available thickening agents via viscosity analysis. The viscosity of the *Dioscorea japonica* liquid was measured according to a measuring method proposed by JDD2013. In addition, the variation in the *Dioscorea japonica* liquid under different conditions was measured. The viscosity of the *Dioscorea japonica* liquid could be easily adjusted by altering its blending and temperature conditions, and its stability against changes in pH and α-amylase activity was similar to those of commercially available thickened liquids. In the presence of NaCl, the viscosity of the commercial thickened liquids decreased significantly depending on the concentration, whereas the viscosity of the *Dioscorea japonica* liquid showed superior stability. Based on the above, the physical properties of the *Dioscorea japonica* liquid in the viscosity analysis were found to be exceptional, and the *Dioscorea japonica* liquid may be suitable as a thickened liquid for patients with dysphagia.

In recent years, xanthan-gum-based thickened liquids have been used, because they are more stable than those based on starch [30]. The two types of thickened liquids used in this study (thickened liquid A and B) were also xanthan-gum-based thickening agents. The two commercially available thickened liquids evaluated in this study mainly contain dextrin and a thickening polysaccharide. However, their viscosity is likely to be different, because the type and the mixing ratio are different depending on the manufacturer [31,32]. Moreover, as with the liquid xanthan gum and commercially available thickened liquids, the *Dioscorea japonica* liquid displayed a positive correlation between the added concentration and the common logarithmic value of viscosity, indicating that the viscosity can be adjusted proportionally to the amount added. The thickened liquids were then evaluated using the LST method, and all the samples were matched with the criteria of the LST value established by JDD2013 (Table 2). The LST method is a simple and inexpensive method for measuring viscosity, with little variation in values from measurement to measurement [26]. Although there are some considerations regarding the reliability of the LST method [33], the LST values of the *Dioscorea japonica* liquid and commercially available thickened liquids in this study were also consistent with the JDD 2013 criteria, as was the viscosity assessment.

Elderly people, particularly those who need meal assistance require more time to eat, and even if a thickening agent is added to warm foods to adjust the viscosity to an appropriate level, the viscosity often changes before eating. Therefore, the viscosity of thickened liquids must be stably maintained under conditions of a change in temperature. Deto et al. reported that the viscosity of liquid xanthan gum does not change significantly from 10 °C to 90 °C [31]. Therefore, it is used as a raw material for commercially available thickened liquids. Nevertheless, in the present study, the viscosity showed a tendency to increase slightly at low or high temperatures (Figure 2a). This is thought to be due to a difference in the determination method of their physical property, i.e., our viscosity analysis and their texture analysis. Hong et al. reported that the viscosity of xanthan-gum-based thickened liquids is temperature-dependent [22], indicating similar results to our present study. Indeed, in the present study, the viscosity of the xanthan-gum-based thickened liquid A showed a significant decrease at high temperatures, whereas the other xanthan-gum-based thickened liquid B was relatively stable in terms of viscosity in response to temperature changes. Although details of the ingredients present in the commercially available thickening agents have not been published, other ingredients have been added to some thickened liquids to improve them, so that their viscosity is not considerably affected by changes in temperature. Compared with these thickened liquids, the *Dioscorea japonica* liquid showed a trend of having a slightly higher viscosity at low temperatures and a slightly lower viscosity at high temperatures. However, this trend was not as significant as that observed for commercially available thickened liquid A. The viscous component of yam, including *Dioscorea japonica*, is derived from glycoproteins [34,35], and the glycoproteins are denatured and insolubilized to reduce their viscosity through heating [36]. Therefore, the viscosity of the *Dioscorea japonica* liquid might be decreased at high temperatures in the present study. Additionally, Miyaoka et al. have reported that the ease of swallowing a thickened liquid varies depending on the temperature [37]. In our previous study, which included a swallowing test of the thickened liquid of *Dioscorea japonica* on subjects, the thickened liquid is similar to commercially available thickened liquids and yogurt at room temperature in terms of suitability [11]. In the future, it is necessary to study, using a swallowing test on subjects, the thickened liquid of *Dioscorea japonica* at various temperatures.

The viscosity stability of the thickened liquid under changes in its pH is crucial for dysphagia diets. As shown in Figure 2b, the viscosity of the *Dioscorea japonica* liquid showed a slightly increasing trend under acidic and alkaline conditions (within +7%). In contrast, the viscosity of the xanthan gum showed a considerable decrease of approximately 30% and 45% at pH 4.0 and 9.0, respectively, and that of thickened liquid B showed a decrease of approximately 15% at pH 9.0. However, since Hadde et al. have reported that xanthan-gum-based thickened liquids were more resistant to pH change [38], we re-experimented with the xanthan gum prepared by their method; however, the results did not show much difference with the present study. Furthermore, the effects of a pH change varied for the two commercially available xanthan-gum-based agents. Yoon et al. reported that pH has different viscoelastic effects depending on the type of thickened liquids [39], and indeed, the present study showed the differences among the xanthan gum and the two commercially available xanthan-gum-based agents. In contrast, the viscosity of the *Dioscorea japonica* liquid was derived from glycoprotein and was shown to be relatively stable with a change in pH.

Considering the addition of thickened liquids to foods containing salt, such as soups, it is crucial that the viscosity is not considerably affected by the addition of salt. In this study, changes in viscosity with the addition of 1% or 5% NaCl were also evaluated and analyzed. It was shown that the viscosity of the liquid xanthan gum and xanthan-gum-based commercially available thickened liquids decreased in proportion to the amount of NaCl added (Figure 3a). The NaCl concentration of a typical soup is approximately 0.8%–0.9%, but even at 1% NaCl, which is close to the above value, a decrease in viscosity of 40%–50% was observed in the liquid xanthan gum and commercially available thickened liquids. The viscosity of xanthan gum, which has a wide variety of structures with many side chains surrounding the main chain [40], stabilizes after the addition of salt at low concentrations (1% or less) [41,42]. However, the present study showed that the addition of 1% or more NaCl caused a decrease in the viscosity of the liquid xanthan gum, indicating that the thickening effect of xanthan gum was affected by the NaCl concentration of the food. In addition, it is known that the addition of NaCl has a notable effect on the rheological properties of xanthan-gum-based thickened liquids [43]. The analysis of the viscosity 2 h and 4 h after the addition of NaCl showed little difference from the present data of Figure 3a. Although the viscosity of the *Dioscorea japonica* liquid increased by approximately 10% with the addition of 5% NaCl, it showed exceptional stability compared with the commercially available thickened liquids; moreover, the *Dioscorea japonica* liquid was barely affected by the addition of 1% NaCl (which is close to the NaCl concentration of a typical soup). In a previous study on tsukuneimo, ichoimo, and nagaimo, which are types of wild yams, the addition of more than 0.1 M (≒0.58%, *W*/*V*) NaCl decreased their viscosity by more than 20% [36]. These results were different in the case of the *Dioscorea japonica* liquid, but the content of the viscous component may vary individually among the yam family [35]. In particular, the stability of the viscosity of the *Dioscorea japonica* liquid against the addition of NaCl is superior in terms of physical properties, and it may be useful in cooking and food processing of swallowing-adjusted foods.

Salivary α-amylase hydrolyzes α-1,4-glycosidic bonds of polysaccharides, such as starch, and immediately decreases the viscosity of food and liquid thickened by starch. However, xanthan gum, which main chain is composed of α-1,4-glycosidic bonds, and xanthan-gum-based commercial thickened liquids are resistant to α-amylase, and their viscosity does not change with the addition of α-amylase. Although the viscous component of *Dioscorea japonica* is different from that of xanthan gum, the *Dioscorea japonica* liquid showed strong resistance to α-amylase, similar to that of liquid xanthan gum or commercial thickened liquids; therefore, the *Dioscorea japonica* liquid would be useful for dysphagia diets (Figure 3b).

In the current study, each liquid was found to have unique characteristics when the change in viscosity of the different thickened liquids was compared.

## 5. Conclusions

Our previous study demonstrated that *Dioscorea japonica* suppressed the proinflammatory lipid mediator PGE_2_ synthetic pathway, which is associated with acute/chronic inflammation [1,2,3] and, additionally, had potential applicability in the development of dysphagia diets [9,11]. In the present study, *Dioscorea japonica* powder showed high versatility, because the viscosity of the *Dioscorea japonica* liquid could be easily adjusted by modifying its content percentages and temperature. In addition, we demonstrated the usefulness of the *Dioscorea japonica* liquid as a thickened liquid, which has excellent suitability compared with commercial thickened liquids. Therefore, we believe that *Dioscorea japonica* liquid would be a novel thickened liquid with adequate food functionality for the prevention of proinflammatory lipid mediator-related diseases.

## Figures and Tables

**Table 1:** Sample concentration at each viscosity level.

Viscosity (mPa∙s)	*Dioscorea japonica* Liquid	Xanthan Gum	Thickened Liquid A	Thickened Liquid B
	(%)	(%)	(%)	(%)
Mildly thick (50–150)	6.22–11.87	<0.93	0.29–1.45	0.02–1.56
Moderately thick (150–300)	11.87–15.44	0.93–1.67	1.45–2.19	1.56–2.53
Extremely thick (300–500)	15.44–18.07	1.67–2.21	2.19–2.73	2.53–3.24

According to Figure 1, the concentration of each sample fitted to the standard viscosity was determined. The solutes were dissolved in distilled water. The viscosity of all samples was measured using a viscometer at 20 °C. Viscosity was categorized into three levels, as established by the Japanese Dysphagia Diet 2013 (JDD2013) [29].

**Table 2:** The standard of viscosity and LST by Japanese Dysphagia Diet 2013 and measured values.

	Mildly Thick	Moderately Thick	Extremely Thick
Standard Viscosity (mPa·s) [21]	50–150	150–300	300–500
Measured Viscosity (mPa·s)			
*Dioscorea japonica* liquid	92.96 ± 0.10	235.90 ± 0.36	401.84 ± 0.93
Xanthan gum	100.18 ± 0.16 *	236.12 ± 0.64	404.58 ± 0.29
Thickened liquid A	93.76 ± 0.52	242.28 ± 0.35	382.54 ± 0.57
Thickened liquid B	95.84 ± 0.20	229.92 ± 0.22	394.28 ± 0.32
Standard LST (mm) [21]	36–43	32–36	30–32
Measured LST (mm)			
*Dioscorea japonica* liquid	39.11 ± 0.93	34.56 ± 0.31	31.39 ± 0.06
Xanthan gum	39.39 ± 0.24	32.83 ± 0.00	31.56 ± 0.56
Thickened liquid A	42.78 ± 0.47 *	34.33 ± 0.48	31.39 ± 0.24
Thickened liquid B	42.06 ± 0.29	34.78 ± 0.24	30.94 ± 0.20

Standard viscosity and LST categorized into three levels, as established by the Japanese Dysphagia Diet 2013 (JDD2013) [21]. *Dioscorea japonica* liquid was used at concentrations of 9.5%, 14%, and 17%. Xanthan gum was used at concentrations of 0.5%, 1.4%, and 2.0%. Thickened liquid A was used at concentrations of 1.0%, 1.8%, and 2.5%. Thickened liquid B was used at concentrations of 1.0%, 2.0%, and 3.0%. The solutes were dissolved in distilled water. Viscosity of each sample was measured using a viscometer at 20 °C in five separate measurements. LST of each sample was measured using a flat plate with concentric circles at 20 °C after three independent measurements. * *p* < 0.01 compared with *Dioscorea japonica*.

## Data Availability

The data used to support the findings of this study can be made available by the corresponding author upon request.
